# Bloqueadores de Canais de Cálcio Não-Diidropiridínicos na Fibrilação Atrial com Fração de Ejeção Levemente Reduzida: Prudência ainda se Impõe

**DOI:** 10.36660/abc.20260195

**Published:** 2026-07-24

**Authors:** Jefferson Luis Vieira, Carlos Eduardo Lucena Montenegro

**Affiliations:** 1 Hospital de Messejana Dr. Carlos Alberto Studart Fortaleza CE Brasil Hospital de Messejana Dr. Carlos Alberto Studart, Fortaleza, CE – Brasil; 2 Universidade Federal do Ceará Fortaleza CE Brasil Universidade Federal do Ceará, Fortaleza, CE – Brasil; 3 Universidade Christus Fortaleza CE Brasil Universidade Christus, Fortaleza, CE – Brasil; 4 Universidade de Pernambuco PROCAPE – Hospital do Coração de Pernambuco Recife PE Brasil PROCAPE – Hospital do Coração de Pernambuco – Universidade de Pernambuco (UPE), Recife, PE – Brasil; 5 Hospital Esperança Recife Recife PE Brasil Hospital Esperança Recife, Recife, PE – Brasil

**Keywords:** Fibrilação Atrial, Insuficiência Cardíaca, Bloqueadores dos Canais de Cálcio

Prezado Editor,

Lemos com atenção o brilhante documento da Diretriz Brasileira de Fibrilação Atrial – 2025 e cumprimentamos seus autores pelo esforço de síntese e pela atualização criteriosa das recomendações para a prática clínica nacional.^1^ Entretanto, desejamos respeitosamente questionar a recomendação referente ao uso de bloqueadores de canais de cálcio não-diidropiridínicos (BCCa-NDHP) para controle de frequência em pacientes com fibrilação atrial (FA) e insuficiência cardíaca com fração de ejeção levemente reduzida (ICFElr). A diretriz corretamente contraindica BCCa-NDHP quando a fração de ejeção do VE (FEVE) <40% e afirma, de forma geral, que não devem ser usados em disfunção sistólica. No entanto, na figura 18, no ramo "Estratégia de Controle da Frequência", os BCCa-NDHP recebem classe de recomendação I em indivíduos com FEVE >40%; isso significa que, pelo fluxograma oficial da Sociedade Brasileira de Cardiologia, pacientes na faixa "cinzenta" da ICFElr (41–49%) ficam incluídos na recomendação de BCCa-NDHP.

O conceito de ICFElr surgiu em 2016, quando a *European Society of Cardiology* (ESC) introduziu formalmente a categoria *Heart Failure with mid-range Ejection Fraction* (HFmrEF, FEVE 40–49%) em sua diretriz de IC. Mais recentemente, a Segunda Definição Universal de Insuficiência Cardíaca refinou esse conceito ao enfatizar que os fenótipos de insuficiência cardíaca não devem ser interpretados exclusivamente por pontos de corte rígidos da FEVE, mas como um contínuo biológico com distintas implicações fisiopatológicas e terapêuticas.^2 , 3^ Antes de 2016, os pacientes com FEVE entre 40–49% eram geralmente incluídos em estudos de ICFEp, o que gerava heterogeneidade significativa nas coortes, mas evidências subsequentes de análises de subgrupos de estudos como o CHARM- Preserved,^4^ TOPCAT,^5^ e PARAGON-HF^6^ reforçaram que pacientes com FEVE no limite inferior da faixa preservada (40–49%) parecem responder melhor a terapias típicas da ICFEr do que aqueles com FE ≥50%. Esse entendimento contemporâneo reforça que pacientes com ICFElr não devem ser simplesmente agrupados àqueles com função sistólica verdadeiramente preservada.

Essa distinção torna-se ainda mais relevante após a publicação da Segunda Definição Universal de Insuficiência Cardíaca, que reconhece explicitamente as limitações inerentes aos pontos de corte rígidos da FEVE. O documento destaca a variabilidade da medida da FEVE, a sobreposição biológica entre os fenótipos e a natureza contínua da disfunção ventricular, tornando inadequado basear decisões terapêuticas exclusivamente em um limiar de FEVE >40%.^3^ O *Multicenter Diltiazem Post-Infarction Trial* (MDPIT)^7^ randomizou mais de 2.400 pacientes pós- infarto para diltiazem ou placebo, com resultado neutro para mortalidade e interação significativa com função ventricular e sinais de congestão: pacientes com congestão pulmonar ou FEVE reduzida (<40%) apresentaram pior prognóstico sob diltiazem, com aumento de IC tardia e mortalidade cardiovascular, enquanto aqueles com FEVE ≥40% não tiveram esse excesso de risco. No entanto, o estudo não avaliou especificamente o intervalo "intermediário" (40–50%). Análises subsequentes demonstraram aumento de IC tardia e eventos em subgrupos com reduções menos acentuadas da FEVE.^8^ Embora contextualizado no período pós-infarto e não em FA isolada, esses dados sustentam o princípio de precaução quando existe inotropismo negativo em ventrículos vulneráveis.

Para controle de frequência, betabloqueadores permanecem primeira linha em IC com qualquer grau de disfunção sistólica; digoxina pode ser útil como adjuvante em repouso e em pacientes hipotensos ou com broncoespasmo. Em muitos casos, estratégias de *early rhythm control* (antiarrítmicos apropriados ao fenótipo e/ou ablação) são preferíveis por potencial impacto em sintomas e desfechos — inclusive em ICFEr. Em ICFElr, onde a reserva contrátil é menor, priorizar controle do ritmo e otimizar Terapia Médica Direcionada por Diretrizes (GDMT) parece mais fisiológico do que adicionar um cronotrópico negativo com efeito inotrópico negativo.^9^ A própria diretriz destaca superioridade do controle do ritmo em diversos cenários.^1^

Recentemente, o iCARDIO *Alliance Global Implementation Guidelines on Heart Failure* 2025, uma iniciativa que visa a implementação global de diretrizes, recomendou explicitamente pela não utilização de BCCa-NDHP quando FEVE <50% por risco de sobrecarga volêmica e piora clínica.^10^ Esse posicionamento também encontra respaldo conceitual na Segunda Definição Universal de Insuficiência Cardíaca, que desencoraja interpretar pacientes com FEVE entre 41–49% como equivalentes àqueles com função ventricular verdadeiramente preservada.

Para reduzir interpretações potencialmente perigosas à beira-leito, na nossa opinião, a medida mais clara seria ajustar a figura da diretriz, substituindo o ponto de corte de 40% por 50%. Dessa forma, pacientes com FEVE entre 41–49% (ICFElr) seriam adequadamente incluídos no grupo em que se deve evitar o uso de verapamil e diltiazem, em consonância com a recomendação textual que já orienta a não utilização em "disfunção sistólica". Essa modificação simples no fluxograma garantiria maior consistência interna do documento e alinhamento com a literatura internacional, além de reduzir interpretações equivocadas na prática clínica ( Figura 1 ).

**Figura 1:**
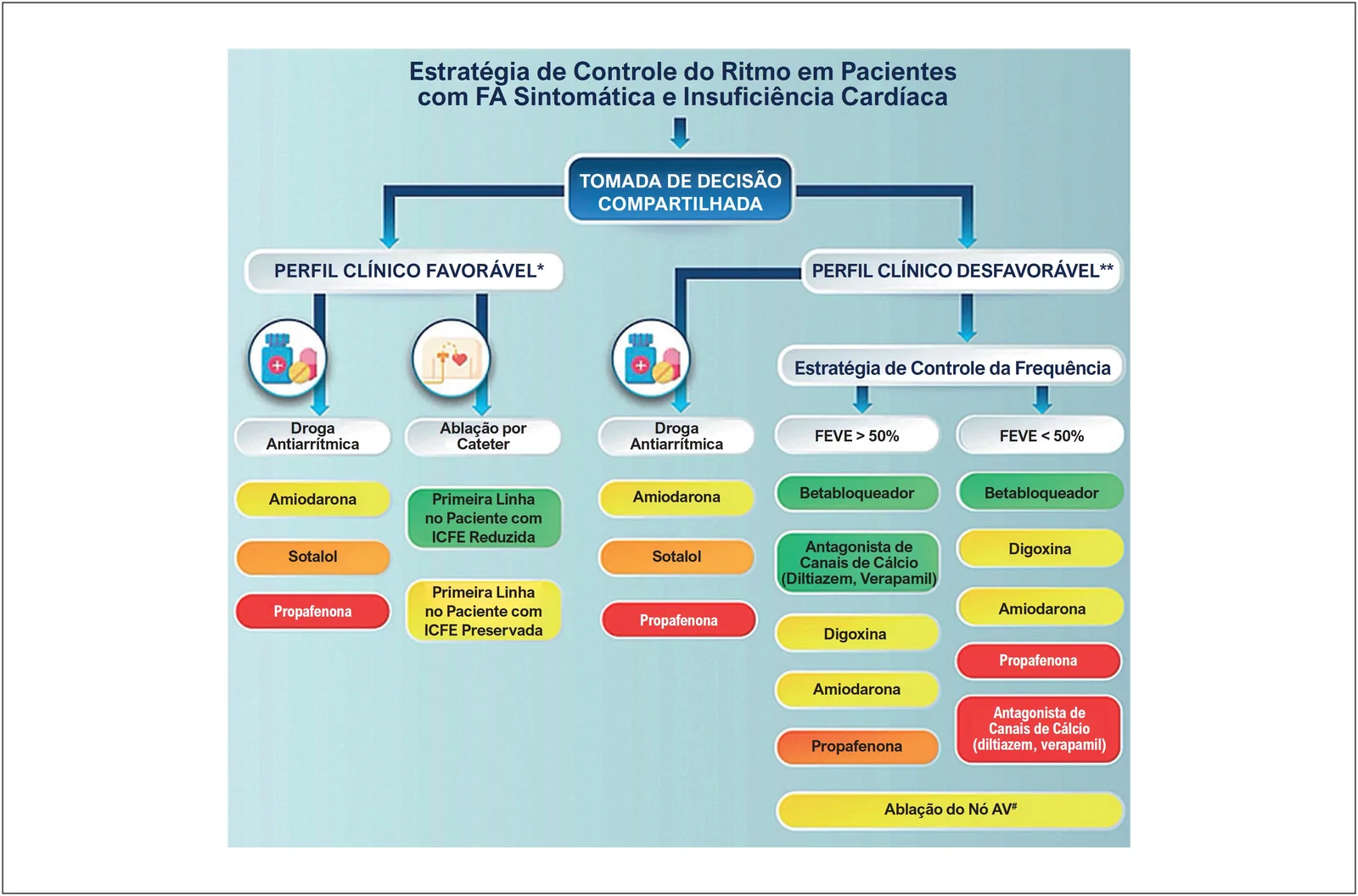
Estratégia de controle do ritmo em pacientes com FA Sintomática e Insuficiência Cardíaca. Fonte: Cintra et al.^1^ *Taquicardiomiopatia, jovens com poucas comorbidades, átrio pouco dilatado, formas paroxísticas ou persistente recente, insuficiência cardíaca inicial, ventrículo com pouca fibrose. **Insuficiência cardíaca avançada, fibrose ventricular, cardiomiopatia fibrótica atrial avançada, recorrência após ablação, muito idoso com várias comorbidades, FA de longa duração. #Ablação do nó AV em casos de falha no controle da frequência e ritmo.

## Conclusão

Dada a ausência de dados robustos de segurança e benefício dos BCCa-NDHP em pacientes com ICFElr e a existência de alternativas alinhadas ao prognóstico, a recomendação atual pode ser interpretada de modo permissivo e induzir a práticas potencialmente deletérias. Além disso, a Segunda Definição Universal de Insuficiência Cardíaca oferece suporte conceitual adicional à presente proposta ao reconhecer a ICFElr como um fenótipo biológico próprio e desencorajar o raciocínio terapêutico baseado exclusivamente em limiares arbitrários da FEVE. Defendemos que a diretriz reforce explicitamente a não recomendação de verapamil/diltiazem quando FEVE <50%, até que estudos específicos em ICFElr validem sua segurança e neutralidade prognóstica.
