# Supplementary material for: Bloqueadores de Canais de Cálcio Não-Diidropiridínicos na Fibrilação Atrial com Fração de Ejeção Levemente Reduzida: Prudência ainda se Impõe
Source: Arq Bras Cardiol. 2026 Jul 24;123(6):e20260195. [Article in Portuguese] doi: 10.36660/abc.20260195 (PMC13668455; doi:10.36660/abc.20260195)
Supplement: Translation

# Translation: Non-dihydropyridine Calcium Channel Blockers in Atrial Fibrillation with Mildly Reduced Ejection Fraction: Caution is Still Advised

**DOI:** 10.36660/abc.20260195i

**Authors:** Jefferson Luis Vieira, Carlos Eduardo Lucena Montenegro

**Affiliations:** 1 Fortaleza CE Brazil Hospital de Messejana Dr. Carlos Alberto Studart, Fortaleza, CE – Brazil; 2 Fortaleza CE Brazil Universidade Federal do Ceará, Fortaleza, CE – Brazil; 3 Fortaleza CE Brazil Universidade Christus, Fortaleza, CE – Brazil; 4 Recife PE Brazil PROCAPE – Hospital do Coração de Pernambuco – Universidade de Pernambuco (UPE), Recife, PE – Brazil; 5 Recife PE Brazil Hospital Esperança Recife, Recife, PE – Brazil

**Keywords:** Atrial Fibrillation, Heart Failure, Calcium Channel Blockers

Dear Editor,

We have carefully read the brilliant document of the Brazilian Atrial Fibrillation Guideline – 2025 and congratulate its authors for their effort in synthesis and for the meticulous updating of the recommendations for national clinical practice.^1^However, we respectfully wish to question the recommendation regarding the use of non-dihydropyridine calcium channel blockers (NDHP-CCBs) for rate control in patients with atrial fibrillation (AF) and heart failure with mildly reduced ejection fraction (HFmrEF). The guideline correctly contraindicates NDHP-CCBs when the left ventricular ejection fraction (LVEF) <40% and states, in general, that they should not be used in systolic dysfunction. However, in Figure 18, in the "Rate Control Strategy" branch, NDHP-CCBs receive a class I recommendation in individuals with LVEF >40%; this means that, according to the official flowchart of the *Sociedade Brasileira de Cardiologia* , patients in the "gray" range of HFmrEF (41–49%) are included in the NDHP-CCB recommendation.

The concept of HFmrEF emerged in 2016, when the European Society of Cardiology (ESC) formally introduced the category Heart Failure with mid-range Ejection Fraction (HFmrEF, LVEF 40–49%) in its HF guideline. More recently, the Second Universal Definition of Heart Failure further refined this concept by emphasizing that heart failure phenotypes should not be interpreted solely through rigid LVEF thresholds, but rather as a biological continuum with distinct pathophysiological and therapeutic implications.^2 , 3^Before 2016, patients with LVEF between 40–49% were generally included in HFpEF studies, which generated significant heterogeneity in cohorts, but subsequent evidence from subgroup analyses of studies such as CHARM-Preserved,^4^TOPCAT,^5^and PARAGON-HF^6^reinforced that patients with LVEF at the lower limit of the preserved range (40–49%) appear to respond better to typical HFrEF therapies than those with EF ≥50%. This contemporary understanding reinforces that patients with mildly reduced EF should not simply be grouped together with those exhibiting truly preserved systolic function.

This distinction has become even more relevant after the publication of the Second Universal Definition of Heart Failure, which explicitly recognizes the inherent limitations of rigid LVEF cutoffs. The document emphasizes that measurement variability, biological overlap between phenotypes, and the continuous nature of ventricular dysfunction make isolated LVEF thresholds an imperfect surrogate for myocardial function. Consequently, therapeutic decisions based exclusively on an LVEF >40% deserve careful reconsideration.^3^The Multicenter Diltiazem Post-Infarction Trial (MDPIT)^7^randomized over 2,400 post-infarction patients to diltiazem or placebo, with a neutral outcome for mortality and a significant interaction with ventricular function and signs of congestion: patients with pulmonary congestion or reduced LVEF (<40%) had a worse prognosis under diltiazem, with increased late HF and cardiovascular mortality, while those with LVEF ≥40% did not have this excess risk. However, the study did not specifically assess the "intermediate" range (40–50%). Subsequent analyses demonstrated increased late HF and events in subgroups with less pronounced reductions in LVEF.^8^Although contextualized in the post-infarction period and not in isolated AF, these data support the precautionary principle when negative inotropism exists in vulnerable ventricles.

For rate control, beta-blockers remain first-line in HF with any degree of systolic dysfunction; digoxin may be useful as an adjunct at rest and in hypotensive patients or those with bronchospasm. In many cases, early rhythm control strategies (antiarrhythmics appropriate to the phenotype and/or ablation) are preferable due to their potential impact on symptoms and outcomes—including in heart failure with reduced ejection fraction (HFrEF). In HFmrEF, where contractile reserve is lower, prioritizing rhythm control and optimizing Guideline-Directed Medical Therapy (GDMT) seems more physiological than adding a negative chronotropic agent with a negative inotropic effect.^9^The guideline itself highlights the superiority of rhythm control in several scenarios.^1^

Recently, the iCARDIO Alliance Global Implementation Guidelines on Heart Failure 2025, an initiative aimed at the global implementation of guidelines, explicitly recommended against the use of NDHP-CCBs when left ventricular ejection fraction (LVEF) <50% due to the risk of volume overload and clinical worsening.^10^Importantly, this recommendation is also conceptually aligned with the Second Universal Definition of Heart Failure, which discourages viewing LVEF 41–49% as equivalent to preserved ventricular systolic function.

To reduce potentially dangerous bedside interpretations, in our opinion, the clearest measure would be to adjust the guideline figure, replacing the 40% cutoff point with 50%. In this way, patients with LVEF between 41–49% (HFmrEF) would be appropriately included in the group where verapamil and diltiazem should be avoided, in line with the textual recommendation that already advises against their use in "systolic dysfunction". This simple modification to the flowchart would ensure greater internal consistency of the document and alignment with international literature, as well as reduce misinterpretations in clinical practice ( Figure 1 ).

**Figure 1:** Rhythm control strategy in patients with symptomatic atrial fibrillation and heart failure. Font: Cintra et al.^1^*Tachycardiomyopathy, young patients with few comorbidities, minimally dilated atrium, paroxysmal or recent persistent forms, early-stage heart failure, ventricle with minimal fibrosis. **Advanced heart failure, ventricular fibrosis, advanced atrial fibrotic cardiomyopathy, recurrence after ablation, very elderly patients with multiple comorbidities, long-standing AF.^#^AV node ablation in cases of failure to control frequency and rhythm.

## Conclusion

Given the lack of robust safety and benefit data for NDHP-CCBs in patients with HFmrEF and the existence of prognostically aligned alternatives, the current recommendation may be interpreted permissively and lead to potentially detrimental practices. Furthermore, the Second Universal Definition of Heart Failure provides additional conceptual support for this proposal by recognizing that patients with mildly reduced EF constitute a distinct biological phenotype and by discouraging therapeutic reasoning based solely on arbitrary LVEF thresholds. We advocate that the guideline explicitly reinforce the non-recommendation of verapamil/diltiazem when LVEF <50%, until specific studies in HFmrEF validate their safety and prognostic neutrality.
