# Supplementary material for: Bloqueadores de Canais de Cálcio Não-Diidropiridínicos na Fibrilação Atrial com Fração de Ejeção Levemente Reduzida: Prudência ainda se Impõe
Source: Arq Bras Cardiol. 2026 Jul 24;123(6):e20260195. [Article in Portuguese] doi: 10.36660/abc.20260195 (PMC13668455; doi:10.36660/abc.20260195)
Supplement: Related texts

# Reply: Carta-resposta

**Authors:** Fatima Dumas Cintra, Cristiano Faria Pisani

**Affiliations:** 1 Universidade Federal de São Paulo São Paulo SP Brasil Universidade Federal de São Paulo, São Paulo, SP – Brasil; 2 Hospital das Clinicas da Faculdade de Medicina da Universidade de São Paulo São Paulo SP Brasil Hospital das Clinicas da Faculdade de Medicina da Universidade de São Paulo, São Paulo, SP – Brasil

Lemos com atenção os comentários criteriosos do autor acerca do uso dos bloqueadores de canais de cálcio não diidropiridínicos na fibrilação atrial com fração de ejeção levemente reduzida e agradecemos o interesse na Diretriz Brasileira de Fibrilação Atrial – 2025.^1^O debate qualificado é fundamental para o aprimoramento contínuo das recomendações e de sua aplicação na prática clínica.

Alguns aspectos merecem ser esclarecidos em relação ao uso dessa classe de medicamentos, ainda frequentemente subutilizada na prática clínica cardiológica. Evidências contemporâneas sugerem que os bloqueadores de canais de cálcio não diidropiridínicos não estão associados à piora de desfechos em pacientes com função ventricular preservada ou levemente reduzida. Uma análise combinada de dados individuais de quatro grandes ensaios clínicos em insuficiência cardíaca, incluindo 16.954 pacientes com FEVE média de 56,8% (79% com FEVE ≥50% e 21% com FEVE entre 40-49%), demonstrou que o uso de bloqueadores de canais de cálcio não esteve associado a aumento nos riscos de morte ou hospitalização por insuficiência cardíaca, sendo inclusive observada redução significativa na mortalidade por falência de bomba (HR 0,76; IC 95% 0,60-0,96).^2^Embora a maioria dos pacientes tenha utilizado diidropiridínicos, o padrão de desfechos foi amplamente similar para bloqueadores de canais de cálcio diidropiridínicos e não diidropiridínicos. Outra série, incluindo apenas pacientes com insuficiência cardíaca, demonstrou que, além de atingir um controle mais rápido da resposta ventricular em comparação com o metoprolol, o diltiazem foi seguro sem efeitos adversos de hipotensão, congestão pulmonar ou queda importante na fração de ejeção.^3^Os clássicos estudos DAVIT II e MDPIT, embora desenhados para avaliar prevenção secundária pós-infarto e não especificamente a segurança dos bloqueadores de canais de cálcio não diidropiridínicos, fornecem evidências indiretas de que o risco desses agentes está relacionado à presença de disfunção sistólica, não havendo evidência de dano em pacientes com fração de ejeção preservada ou levemente reduzida.^4 , 5^

Além disso, o ponto de corte adotado na Diretriz Brasileira de Fibrilação Atrial – 2025 está alinhado às principais diretrizes internacionais contemporâneas. As diretrizes da *European Society of Cardiology,* ^6^da ACC/AHA/ACCP/HRS^7^e da Canadian Cardiovascular Society^8^utilizam o limiar de FEVE de 40% para orientar o uso de bloqueadores de canais de cálcio não diidropiridínicos no controle de frequência, não havendo respaldo consistente para a adoção de um ponto de corte mais restritivo de 50%.

Reconhecemos, conforme destacado pelo autor, que o grupo com FEVE entre 41 a 49% representa uma zona de transição fisiopatológica, com características que podem se aproximar tanto da insuficiência cardíaca com fração de ejeção reduzida quanto da preservada. À luz dessas particularidades, o controle do ritmo é a estratégia preferencial, dado seu impacto na redução da mortalidade.^9^Ainda assim, as diretrizes têm como propósito orientar a prática com base na melhor evidência disponível; entretanto, em cenários intermediários ou de incerteza, o julgamento clínico individual permanece insubstituível.

## References

[1] Cintra FD, Pisani CF, Rezende AGDS, Henz BD, Armaganijan LV, Pimentel M (2025). Brazilian Atrial Fibrillation Guidelines - 2025. Arq Bras Cardiol.

[2] Matsumoto S, Kondo T, Yang M, Campbell RT, Docherty KF, Boer RA (2023). Calcium Channel Blocker Use and Outcomes in Patients with Heart Failure and Mildly Reduced and Preserved Ejection Fraction. Eur J Heart Fail.

[3] Compagner CT, Wysocki CR, Reich EK, Zimmerman LH, Holzhausen JM (2022). Intravenous Metoprolol versus Diltiazem for Atrial Fibrillation with Concomitant Heart Failure. Am J Emerg Med.

[4] Goldstein RE, Boccuzzi SJ, Cruess D, Nattel S (1991). Diltiazem Increases Late-Onset Congestive Heart Failure in Postinfarction Patients with Early Reduction in Ejection Fraction. The Adverse Experience Committee; and the Multicenter Diltiazem Postinfarction Research Group. Circulation.

[5] Effect of Verapamil on Mortality and Major Events after Acute Myocardial Infarction (the Danish Verapamil Infarction Trial II--DAVIT II) (1990). Am J Cardiol.

[6] Van Gelder IC, Rienstra M, Bunting KV, Casado-Arroyo R, Caso V, Crijns HJGM (2024). 2024 ESC Guidelines for the Management of Atrial Fibrillation Developed in Collaboration with the European Association for Cardio-Thoracic Surgery (EACTS). Eur Heart J.

[7] Joglar JA, Chung MK, Armbruster AL, Benjamin EJ, Chyou JY, Cronin EM (2024). 2023 ACC/AHA/ACCP/HRS Guideline for the Diagnosis and Management of Atrial Fibrillation: A Report of the American College of Cardiology/American Heart Association Joint Committee on Clinical Practice Guidelines. Circulation.

[8] Andrade JG, Aguilar M, Atzema C, Bell A, Cairns JA, Cheung CC (2020). The 2020 Canadian Cardiovascular Society/Canadian Heart Rhythm Society Comprehensive Guidelines for the Management of Atrial Fibrillation. Can J Cardiol.

[9] Pasqualotto E, Ternes CMP, Chavez MP, Polanczyk CA, Ferreira ROM, Nienkötter T (2024). Catheter Ablation for Atrial Fibrillation in Heart Failure with Reduced Ejection Fraction Patients: A Meta-Analysis. Heart Rhythm.

# Reply

**Authors:** Fatima Dumas Cintra, Cristiano Faria Pisani

**Affiliations:** 1 São Paulo SP Brazil Universidade Federal de São Paulo, São Paulo, SP – Brazil; 2 São Paulo SP Brazil Hospital das Clinicas da Faculdade de Medicina da Universidade de São Paulo, São Paulo, SP – Brazil

We have carefully read the author's insightful comments regarding the use of non-dihydropyridine calcium channel blockers in atrial fibrillation with mildly reduced ejection fraction and appreciate the interest shown in the Brazilian Atrial Fibrillation Guideline – 2025.^1^Qualified debate is essential for the continuous improvement of recommendations and their application in clinical practice.

Several aspects deserve clarification regarding the use of this class of medications, which is still frequently underutilized in cardiology clinical practice. Contemporary evidence suggests that non-dihydropyridine calcium channel blockers are not associated with worsening outcomes in patients with preserved or mildly reduced ventricular function. A combined analysis of individual data from four large clinical trials in heart failure, including 16,954 patients with a mean LVEF of 56.8% (79% with LVEF ≥50% and 21% with LVEF between 40-49%), demonstrated that the use of calcium channel blockers was not associated with increased risks of death or hospitalization for heart failure, and a significant reduction in mortality from pump failure was even observed (HR 0.76; 95% CI 0.60-0.96).^2^Although most patients used dihydropyridines, the pattern of outcomes was largely similar for dihydropyridine and non-dihydropyridine calcium channel blockers. Another series, including only patients with heart failure, demonstrated that, in addition to achieving faster ventricular response control compared to metoprolol, diltiazem was safe without adverse effects of hypotension, pulmonary congestion, or a significant drop in ejection fraction.^3^The classic DAVIT II and MDPIT studies, although designed to evaluate secondary prevention after myocardial infarction and not specifically the safety of non-dihydropyridine calcium channel blockers, provide indirect evidence that the risk of these agents is related to the presence of systolic dysfunction, with no evidence of harm in patients with preserved or slightly reduced ejection fraction.^4 , 5^

Furthermore, the cutoff point adopted in the Brazilian Atrial Fibrillation Guideline – 2025 is aligned with the main contemporary international guidelines. These guidelines are from the European Society of Cardiology,^6^from ACC/AHA/ACCP/HRS,^7^and the Canadian Cardiovascular Society^8^use an LVEF threshold of 40% to guide the use of non-dihydropyridine calcium channel blockers in rate control, and there is no consistent support for adopting a more restrictive cutoff point of 50%.

As highlighted by the author, we recognize that the group with LVEF between 41 and 49% represents a pathophysiological transition zone, with characteristics that may resemble both heart failure with reduced ejection fraction and preserved ejection fraction. In light of these particularities, rhythm control is the preferred strategy, given its impact on reducing mortality.^9^Even so, guidelines aim to guide practice based on the best available evidence; however, in intermediate or uncertain scenarios, individual clinical judgment remains irreplaceable.
